# Como Demonstrar o Benefício Prognóstico do Rastreamento Agressivo da Fibrilação Atrial

**DOI:** 10.36660/abc.20250138

**Published:** 2025-08-06

**Authors:** Naoya Kataoka, Teruhiko Imamura

**Affiliations:** 1 University of Toyama Toyama Japão University of Toyama, Toyama – Japão

**Keywords:** Arritmias Cardíacas, Dispositivos Eletrônicos Vestíveis, Acidente Vascular Cerebral

**Ao Editor**

A detecção precoce da fibrilação atrial (FA) assintomática é considerada essencial para a prevenção de eventos tromboembólicos e da progressão da insuficiência cardíaca. Inúmeros dispositivos vestíveis foram desenvolvidos para facilitar esse objetivo. Os autores utilizaram a análise de risco de AVC (SRA), um método que estratifica o risco de FA paroxística por meio da avaliação de dados de eletrocardiograma.^1^ Esse algoritmo se mostrou eficaz no rastreamento de pacientes com FA paroxística assintomática, com 29,4% dos participantes categorizados como de alto risco. Entre esses indivíduos de alto risco, a FA foi detectada em 38,7% após um período de rastreamento de 7 dias. No entanto, várias questões críticas merecem discussão.

No estudo atual, 67,4% dos participantes foram classificados como de baixo risco para FA.^1^ Para avaliar a validade geral do algoritmo SRA, a incidência de FA nessa coorte de baixo risco também deveria ter sido avaliada por meio de monitoramento de 7 dias. Dado que o estudo incluiu pacientes com 65 anos ou mais, é plausível que alguns indivíduos classificados como de baixo risco possam ter apresentado episódios de FA não detectados.^2^

A duração ideal para o rastreamento de FA permanece incerta. Períodos de monitoramento prolongados provavelmente melhorarão as taxas de detecção de FA.^3^ É necessário esclarecer a justificativa por trás da escolha dos autores de um período de rastreamento de 7 dias. Além disso, uma análise de custo-efetividade deve ser conduzida para determinar se a estratificação de risco usando o algoritmo SRA é mais vantajosa em comparação ao monitoramento prolongado do eletrocardiograma em todos os participantes. 0000-0002-1265-1930

Embora o rastreamento intensivo de FA por meio de dispositivos vestíveis, como demonstrado no presente estudo,^1^ pareça aumentar as taxas de detecção, nenhuma evidência conclusiva atualmente sustenta o benefício prognóstico de tais intervenções. Portanto, ensaios clínicos randomizados em larga escala são urgentemente necessários para comparar os resultados dos programas de triagem de FA com o tratamento padrão, elucidando assim o significado clínico da detecção sistemática de FA.
