# Supplementary material for: Como Demonstrar o Benefício Prognóstico do Rastreamento Agressivo da Fibrilação Atrial
Source: Arq Bras Cardiol. 2025 Aug 6;122(7):e20250138. [Article in Portuguese] doi: 10.36660/abc.20250138 (PMC12695098; doi:10.36660/abc.20250138)
Supplement: Translation

# Translation: How to Demonstrate the Prognostic Benefit of Aggressive Screening of Atrial Fibrillation

**DOI:** 10.36660/abc.20250138i

**Authors:** Naoya Kataoka, Teruhiko Imamura

**Affiliations:** 1 Toyama Toyama Japan University of Toyama, Toyama – Japan

**Keywords:** Cardiac Arrhythmias, Wearable Electronic Devices, Stroke

**To Editor**

The early detection of asymptomatic atrial fibrillation (AF) is considered essential for preventing thromboembolic events and the progression of heart failure. Numerous wearable devices have been developed to facilitate this objective. The authors employed stroke risk analysis (SRA), a method that stratifies the risk of paroxysmal AF through the evaluation of electrocardiogram data.^1^This algorithm proved effective in screening patients with asymptomatic paroxysmal AF, with 29.4% of participants categorized as high-risk. Among these high-risk individuals, AF was detected in 38.7% following a 7-day screening period. Nevertheless, several critical concerns merit discussion.

In the current study, 67.4% of participants were classified as low-risk for AF.^1^To assess the overall validity of the SRA algorithm, the incidence of AF within this low-risk cohort should also have been evaluated through 7-day monitoring. Given that the study included patients aged 65 years and older, it is plausible that some individuals classified as low-risk might have experienced undetected AF episodes.^2^

The optimal duration for AF screening remains uncertain. Extended monitoring periods are likely to improve AF detection rates.^3^Clarification on the rationale behind the authors’ choice of a 7-day screening period is warranted. Additionally, a cost-effectiveness analysis should be conducted to determine whether risk stratification using the SRA algorithm is more advantageous compared to prolonged electrocardiogram monitoring across all participants.

Although intensive AF screening via wearable devices, as demonstrated in the present study,^1^appears to enhance detection rates, no conclusive evidence currently supports the prognostic benefit of such interventions. Therefore, large-scale, randomized controlled trials are urgently needed to compare the outcomes of AF screening programs against standard care, thereby elucidating the clinical significance of systematic AF detection.
