# Supplementary material for: Como Demonstrar o Benefício Prognóstico do Rastreamento Agressivo da Fibrilação Atrial
Source: Arq Bras Cardiol. 2025 Aug 6;122(7):e20250138. [Article in Portuguese] doi: 10.36660/abc.20250138 (PMC12695098; doi:10.36660/abc.20250138)
Supplement: Related texts

# Reply: Carta-resposta

**Authors:** Roberto Dischinger Miranda, Weimar Kunz Sebba Barroso

**Affiliations:** 1 Universidade Federal de Sao Paulo Escola Paulista de Medicina São Paulo SP Brasil Universidade Federal de Sao Paulo Escola Paulista de Medicina, São Paulo, SP – Brasil; 2 Universidade Federal de Goiás Liga de Hipertensão Arterial Goiânia GO Brasil Universidade Federal de Goiás – Liga de Hipertensão Arterial, Goiânia, GO – Brasil

Agradecemos profundamente aos autores pelo interesse e importantes comentários relativos ao nosso estudo "Programa para Otimizar a Detecção da Fibrilação Atrial Paroxística: Estudo RITMO".^1^Reconhecemos a relevância das questões levantadas e aproveitamos esta oportunidade para esclarecer pontos fundamentais.

A detecção precoce da fibrilação atrial (FA) assintomática é realmente um tema de grande relevância, e está relacionada à prevenção potencial de eventos, como os tromboembólicos e insuficiência cardíaca (IC).^2^

Em relação à estratificação de risco utilizando a análise de risco de acidente vascular cerebral (SRA, *Stroke Risk Analysis* ), nosso estudo demonstrou que 29,4% dos participantes foram classificados como de alto risco, com uma taxa de detecção de FA de 38,7% após o período de sete dias com eletrocardiogramas domiciliares. A falta de uma ferramenta de estratificação, poderia comprometer os resultados do estudo.^3^Além disso, o SRA demonstrou ser uma ferramenta valiosa para identificar pacientes com maior probabilidade de apresentar FA paroxística.^4 , 5^

Concordamos em que a incidência de FA no grupo classificado como de baixo risco é uma questão muito pertinente. No entanto, nosso estudo teve como objetivo avaliar uma estratégia simples e acessível para otimizar a identificação da FA em pacientes assintomáticos com 65 anos ou mais, portadores de hipertensão arterial ou IC. Além da realização de um monitoramento prolongado em todos os participantes não ser o objetivo primário de nosso estudo, este aumentaria muito o custo global do projeto. Estudos futuros poderiam explorar essa questão, avaliando a real incidência de FA nesse subgrupo ao longo de um período de monitoramento mais extenso.

A escolha do período de sete dias para a monitorização domiciliar foi baseada em estudos prévios.^3^De fato, períodos mais longos poderiam aumentar ainda mais a taxa de detecção,^6^mas devem ser ponderados em relação à adesão do paciente, custo-efetividade e viabilidade clínica. Uma análise específica de custo-efetividade para comparar estratégias alternativas está em consideração para pesquisas futuras.

Concordamos também em que a relevância clínica da triagem sistemática de FA ainda deve ser confirmada por novos ensaios randomizados de larga escala. No entanto, a identificação precoce da FA, especialmente em populações de maior risco, pode permitir a implementação mais precoce de estratégias terapêuticas, incluindo anticoagulação, reduzindo a incidência de eventos tromboembólicos.^7 – 9^

Agradecemos novamente os comentários valiosos e esperamos que nosso estudo contribua para o aprimoramento das estratégias de triagem da FA na prática clínica.

## References

[1] Andrade RP, Vitorino PVO, Sousa ALL, Miranda RD, Nogueira BAA, Cestário EDES (2024). A Program to Optimize the Detection of Paroxysmal Atrial Fibrillation: The RITMO Study. Arq Bras Cardiol.

[2] Freedman B, Camm J, Calkins H, Healey JS, Rosenqvist M, Wang J (2017). Screening for Atrial Fibrillation: A Report of the AF-SCREEN International Collaboration. Circulation.

[3] Lubitz SA, Atlas SJ, Ashburner JM, Lipsanopoulos ATT, Borowsky LH, Guan W (2022). Screening for Atrial Fibrillation in Older Adults at Primary Care Visits: VITAL-AF Randomized Controlled Trial. Circulation.

[4] Schaefer JR, Leussler D, Rosin L, Pittrow D, Hepp T (2014). Improved Detection of Paroxysmal Atrial Fibrillation Utilizing a Software-Assisted Electrocardiogram Approach. PLoS One.

[5] Rizos T, Güntner J, Jenetzky E, Marquardt L, Reichardt C, Becker R (2012). Continuous Stroke Unit Electrocardiographic Monitoring versus 24-Hour Holter Electrocardiography for Detection of Paroxysmal Atrial Fibrillation after Stroke. Stroke.

[6] Goldenthal IL, Sciacca RR, Riga T, Bakken S, Baumeister M, Biviano AB (2019). Recurrent Atrial Fibrillation/Flutter Detection after Ablation or Cardioversion Using the AliveCor KardiaMobile Device: iHEART Results. J Cardiovasc Electrophysiol.

[7] Svennberg E, Friberg L, Frykman V, Al-Khalili F, Engdahl J, Rosenqvist M (2021). Clinical Outcomes in Systematic Screening for Atrial Fibrillation (STROKESTOP): A Multicentre, Parallel Group, Unmasked, Randomised Controlled Trial. Lancet.

[8] Shoamanesh A, Field TS, Coutts SB, Sharma M, Gladstone D, Hart RG (2025). Apixaban versus Aspirin for Stroke Prevention in People with Subclinical Atrial Fibrillation and a History of Stroke or Transient Ischaemic Attack: Subgroup Analysis of the ARTESiA Randomised Controlled Trial. Lancet Neurol.

[9] McIntyre WF, Benz AP, Becher N, Healey JS, Granger CB, Rivard L (2024). Direct Oral Anticoagulants for Stroke Prevention in Patients with Device-Detected Atrial Fibrillation: A Study-Level Meta-Analysis of the NOAH-AFNET 6 and ARTESiA Trials. Circulation.

# Reply

**Authors:** Roberto Dischinger Miranda, Weimar Kunz Sebba Barroso

**Affiliations:** 1 São Paulo SP Brazil Universidade Federal de Sao Paulo Escola Paulista de Medicina,1 São Paulo, SP - Brasil; 2 Goiânia GO Brazil Universidade Federal de Goiás – Liga de Hipertensão Arterial, Goiânia, GO – Brazil

We are deeply grateful to the authors for their interest and valuable comments regarding our study, "A Program to Optimize the Detection of Paroxysmal Atrial Fibrillation: The RITMO Study."^1^We acknowledge the relevance of the issues raised and would like to take this opportunity to clarify key points.

Early detection of asymptomatic atrial fibrillation (AF) is indeed a topic of great relevance, and is related to the potential prevention of events such as thromboembolic events and heart failure (HF).^2^

Regarding risk stratification using Stroke Risk Analysis (SRA), our study demonstrated that 29.4% of participants were classified as high risk, with an AF detection rate of 38.7% after seven days with home electrocardiograms. The lack of a stratification tool could compromise the results of the study.^3^Furthermore, the SRA has been shown to be a valuable tool for identifying patients with a higher probability of paroxysmal AF.^4 , 5^

We agree that the incidence of AF in the group classified as low risk is a very pertinent question. However, our study aimed to evaluate a simple and accessible strategy to optimize the identification of AF in asymptomatic patients aged 65 years or older with arterial hypertension or HF. In addition to performing prolonged monitoring in all participants, which is not the primary objective of our study, this would greatly increase the overall cost of the project. Future studies could explore this issue by assessing the true incidence of AF in this subgroup over a longer monitoring period.

The choice of seven days for home monitoring was based on previous studies.^3^In fact, longer periods could further increase the detection rate,^6^but they should be weighed against patient adherence, cost-effectiveness, and clinical feasibility. A specific cost-effectiveness analysis to compare alternative strategies is under consideration for future research.

We also agree that the clinical relevance of systematic AF screening remains to be confirmed by new large-scale randomized trials. However, early identification of AF, especially in higher-risk populations, may allow earlier implementation of therapeutic strategies, including anticoagulation, reducing the incidence of thromboembolic events.^7 – 9^

We again thank you for your valuable comments and hope that our study will contribute to the improvement of AF screening strategies in clinical practice.
